# Role of Charlson comorbidity index in predicting the ICU admission in patients with thoracic aortic aneurysm undergoing surgery

**DOI:** 10.1186/s13018-023-04364-6

**Published:** 2023-11-16

**Authors:** Yu-fei Zhan, Feng Li, Long-chuan Wu, Jun-ming Li, Can-yan Zhu, Ming-shuai Han, Yi Sheng

**Affiliations:** Emergency Medicine, Linping Campus, The Second Affiliated Hospital of Zhejiang University School of Medicine, No.369 Yingbin Road, Nanyuan Street, Linping District, Hangzhou, 311100 Zhejiang China

**Keywords:** Charlson comorbidity index, TAA, ICU admission, Prediction

## Abstract

**Objectives:**

This study aimed to explore the value of the Charlson comorbidity index (CCI) in predicting ICU admission in patients with aortic aneurysm (AA).

**Methods:**

The clinical data of patients were obtained from the Medical Information Mart for Intensive Care-IV database. The association between CCI and ICU admission was explored by restricted cubic spline (RCS), threshold effect analysis, generalized linear model, logistic regression, interaction, and mediation analyses. Its clinical value was evaluated by decision curve analysis (DCA), receiver operating characteristic curve (ROC), DeLong's test, and net reclassification index (NRI) analyses.

**Results:**

The ICU admission was significantly associated with the thoracic AA (TAA), unruptured status, and surgery status. Therefore, 288 candidate patients with unruptured TAA who received surgery were enrolled in the further analysis. We found that CCI was independently associated with the ICU admission of candidates (*P* = 0.005). Further, their nonlinear relationship was observed (adjusted *P* = 0.008), and a significant turning point of 6 was identified. The CCI had a favorable performance in predicting ICU admission (area under curve = 0.728) and achieved a better clinical net benefit. New models based on CCI significantly improved the accuracy of prediction. Besides the importance of CCI in ICU admission, CCI also exerted important interaction effect (rather than mediating effects) on the association of other variables (such as age and blood variables) with ICU admission requirements (all *P* < 0.05).

**Conclusions:**

The CCI is an important predictor of ICU admission after surgery in patients with unruptured TAA.

## Introduction

Aortic aneurysm (AA) is the second most prevalent aortic disease after atherosclerosis and becomes the ninth-leading cause of death globally (1). AA mainly includes abdominal aortic aneurysm (AAA) and thoracic aortic aneurysm (TAA). Presently, surgical procedure remains the main choice for the treatment of AA. Open surgery repair (OSR) and endovascular aortic repair (EVAR) are the most common methods involved in AA treatment, especially the EVAR is used more often due to its superiority to some extent (2). Because of significant morbidity and mortality, many patients undergoing complex aortic surgical procedures were traditionally admitted to the intensive care unit (ICU) for the 1st postoperative day for close continuous monitoring and supportive therapies (3). Angela et al. study indicated that ICU admission within 24 h after noncardiac surgery occurred in 9.6% of patients (total 541,524), and these patients showed higher median age and burden of prehospital comorbidities (Charlson comorbidity index, CCI ≥ 2) (4). They also found that the proportions of patients admitted to ICU were significantly related to the surgery types, and hysterectomy accounted for 0.9% as well as 90.8% for open AAA repair. However, Lawlor et al. showed that for the majority of elective infrarenal AAA patients undergoing open repair, postoperative ICU admission was unnecessary (5), and these patients can be safely admitted to the stepdown unit for postoperative management. Over 300 million surgical procedures are performed worldwide each year, and ICU resources become more limited, increasing constraints have been placed on ICU admissions.

Therefore, early recognition and management contribute to effective ICU utilization (6). A key strategy for improving the management of ICU is to identify the subgroup of patients who are at high risk of adverse outcomes. At present, several tools have been used to determine which patients require ICU admission, such as quick Sequential Organ Failure Assessment (qSOFA) score, Systemic Inflammatory Response Syndrome (SIRS), and National Early Warning Score (NEWS). Stokes et al. indicated that a preoperative qSOFA score ≥ 2 can predict postoperative ICU admission in patients with obstructed infected ureteral stones (7). Goulden et al. showed that for predicting the ICU admission of patients with sepsis, the performance of sensitivity was NEW = SIRS > qSOFA and qSOFA > NEW > SIRS for specificity (8). It should be noted that no scoring system has both high sensitivity and specificity for predicting adverse outcomes. Hence, it was necessary to find a more useful tool to predict ICU admission, which contributes to the management of ICU resources and decreases health-care costs.

Up to now, the previous studies only indicated that a high burden of prehospital comorbidities and AAA surgery showed a higher rate of ICU admission. However, no study reported a detailed association between CCI and ICU admission in patients with AA. In this study, we found that the patients with unruptured TAA undergoing the surgery, rather than AAA, were significantly related to the need for ICU admission. Therefore, we performed a series of analyses to evaluate the relationship between CCI of patients with TAA and ICU admission and the potential influence of CCI on the association of other significant variables with ICU admission. We also explored the clinical value of CCI to predict ICU admission in patients with TAA.

## Methods

### Data source and study population

In this study, the Medical Information Mart for Intensive Care-IV (MIMIC-IV), a publicly available single-center critical care database, was used to obtain the data. The platform can be accessed after finishing a web course offered by the National Institutes of Health (NIH).

In this study, these patients were enrolled in the further analysis: diagnosed with abdominal aortic aneurysm (AAA, ruptured and unruptured) and thoracic aortic aneurysm (TAA, ruptured and unruptured); had ICU data; had surgery records; aged ≥ 18 years old; and length of in-hospital stay ≥ 2 days. The patients missing Charlson comorbidity index (CCI) data were excluded. For patients with multiple admissions records, we only retained the record on the patient’s first admission to the ICU.

### Outcomes and covariables

In this study, the outcome was the ICU requirement of patients with AA. The primary independent variable was the CCI. The demographical covariables included age, gender, marital status (single, married, divorced, and widowed), and body mass index (BMI). The BMI was grouped into four groups, including underweight (BMI < 18.5 kg/m^2^), normal weight (18.5–24.9 kg/m^2^), overweight (25.0–29.9 kg/m^2^), and obese (≥ 30.0 kg/m^2^). We also obtained the histories of hypertension, coronary heart disease (CHD), hyperlipidemia, and chronic obstructive pulmonary disease (COPD). In addition, these data within 24 h after admission, including systolic blood pressure (SBP), diastolic blood pressure (DBP), mean arterial pressure (MAP), platelet (K/μL), urea nitrogen (mg/dL), creatinine (mg/dL), anion gap (mEq/L), and red cell volume distribution width (RDW, %), were also collected.

### Statistical analysis

The association between CCI and ICU requirements of patients with AA was first explored by logistic regression analysis based on aortic aneurysm type (AAA and TAA), disease progression (ruptured and unruptured), and surgery status (with and without). According to these results, the candidate patients for further analysis were determined. Baseline characteristics of final candidate patients were presented stratified by the ICU requirement (yes vs no). The continuous variables conforming to the normal distribution were presented as the means ± SD, and the independent t-test was used to compare the difference between the two groups. Whereas the median and quartiles were used for the nonnormal variables, and the Mann–Whitney U-test was used to compare the differences. Categorical variables were expressed as frequencies, and the distribution difference was analyzed by χ^2^ test.

Then, two models were established to explore the independent association between CCI and ICU admission requirements. Firstly, the restricted cubic spline (RCS) was performed to assess their association. The knots for RCS were set as 5. According to the book Regression Modeling Strategies by F. E. Harrell, the number of knots = 5 is a good choice when the sample size is > 100. To determine the significant reference point for RCS analysis, the threshold effect analysis was performed, and a significant inflection point affecting their association was obtained. Subsequently, the inflection point was set as the reference point in RCS analysis. Further, generalized linear model was performed to assess their association. In addition, we transformed the data of CCI as the log-CCI, and a linear relationship between log-CCI and ICU admission requirements was observed. Hence, the association between log-CCI and ICU admission requirements was also explored by the logistic regression analysis. The interaction effect of other variables on CCI was also evaluated both in two models. Next, we explored the clinical value of CCI for predicting the ICU requirement by decision curve analysis (DCA) and receiver operating characteristic curve (ROC) analysis. Based on CCI, we established three new models to predict the ICU requirement by introducing other variables. The DeLong's test and net reclassification index (NRI) analysis were performed to compare the performance of three models. In addition, we also assessed the potential role of CCI as the interaction factor and mediating factor on the association of other variables with ICU requirements by interaction and mediating analyses. All the data were analyzed using the SPSS and R software. *P* < 0.05 was considered statistically significant.

## Results

### The research participants

In this study, a total of 1180 patients with aortic aneurysms were found. We first explored the association between CCI and ICU requirement (yes vs no) through subgroup analysis stratified by aortic aneurysm type, disease progression, and surgery status. The results (Table 1) showed that a significant association between them can be found in patients with TAA (OR = 0.771, *P* < 0.001), unruptured status (OR = 0.871, *P* < 0.001), and those who underwent the surgery (OR = 0.876, *P* < 0.001). Therefore, 288 patients with unruptured TAA who received the surgery were finally enrolled in the further analysis. Of whom, 226 patients (78.5%) had a record of ICU admission.

**Table 1:** The association analysis between CCI and ICU requirements in patients with AA

		OR (95%CI)	P
Aortic aneurysm type	AAA (n = 770)	0.957 [0.903, 1.014]	0.137
	TAA (n = 410)	0.771 [0.707, 0.841]	< 0.001
Disease progression	Ruptured (n = 40)	0.574 [0.293, 1.123]	0.105
	Unruptured (n = 1140)	0.871 [0.831, 0.914]	< 0.001
Surgery status	Yes (n = 881)	0.876 [0.829, 0.927]	< 0.001
	No (n = 299)	0.989 [0.888, 1.101]	0.838

It should be noted that among 288 patients with TAA, 12 patients had alcohol data; seven patients had smoking data; two patients had a history of diabetes; and seven patients had renal disease history. The samples with these variables were too small, which limited our statistical analysis. Therefore, these variables were not included in our final analysis.

The characteristics of patients with TAA are presented in Table 2. The results showed that there were differences between ICU and non-ICU groups in terms of marital status (*P* = 0.029), age (*P* = 0.009), CCI (*P* < 0.001), the levels of platelet (*P* = 0.008), urea nitrogen (*P* < 0.001), creatinine (*P* < 0.001), and RDW (*P* = 0.001). These six variables with statistical significance were selected for further analyses.

**Table 2:** The clinical characteristics of patients with TAA based on the ICU requirements

		Non-ICU (n = 62)	ICU (n = 226)	Statistics	P
Gender	Male	37	153	1.395	0.238
	Female	25	73		
BMI category	Underweight	1	7	3.665	0.300
	Normal	12	37		
	Overweight	23	43		
	Obese	26	83		
Marital status	Single	15	49	9.022	0.029
	Married	28	122		
	Divorced	4	23		
	Widowed	13	18		
Hypertension	No	26	92	0.030	0.862
	Yes	36	134		
CHD	No	40	161	1.043	0.307
	Yes	22	65		
Hyperlipidemia	No	28	111	0.305	0.581
	Yes	34	115		
COPD	No	51	197	0.981	0.322
	Yes	11	29		
Age	Years	70.0 ± 1.5	64.9 ± 0.9	2.629	0.009
SBP	mmHg	127.9 ± 2.3	130.1 ± 1.3	− 0.736	0.462
DBP	mmHg	72.4 ± 1.7	74.0 ± 0.9	− 0.780	0.432
MAP	mmHg	90.9 ± 1.5	92.7 ± 0.9	− 0.892	0.436
CCI	–	7 [6,9]	5 [4,6]	− 5.575	< 0.001
Platelet	K/μL	189 [146, 244]	158 [120, 206]	− 2.654	0.008
Urea nitrogen	mg/dL	21 [15, 30]	16 [13, 20]	− 4.357	< 0.001
Creatinine	mg/dL	1.1 [0.8, 1.5]	0.9 [0.7, 1.1]	− 3.563	< 0.001
Anion gap	mEq/L	13 [12, 15.5]	13 [11, 15]	− 1.806	0.071
RDW	%	14.1 [13.3, 16.1]	13.5 [12.8, 14.6]	− 3.430	0.001
Length of stay	Days	3.9 [2.8, 8.1]	6.6 [5.2, 9.9]	− 4.815	< 0.001

### Association between CCI and ICU admission requirement

**Fig. 1:**
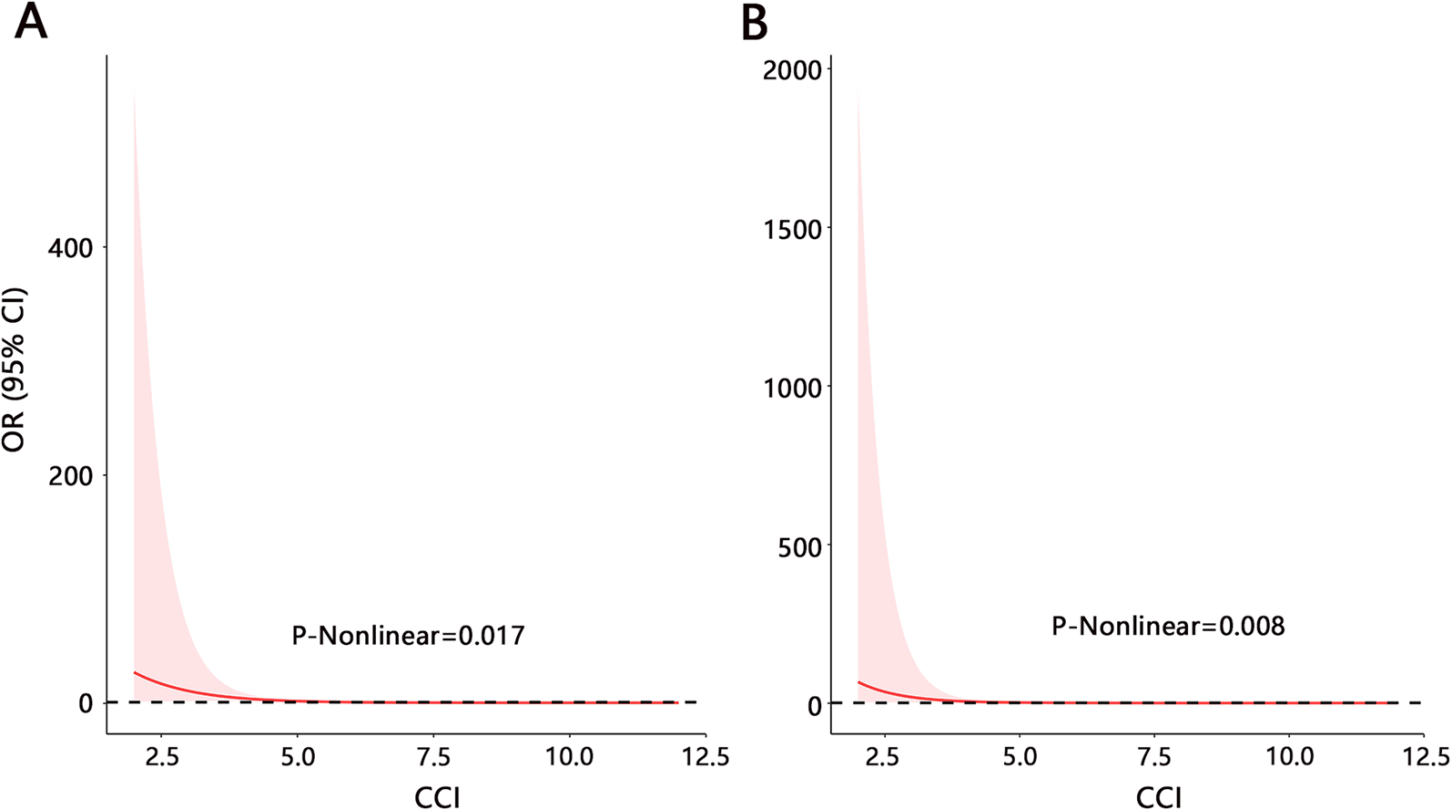
The association visualization between CCI and ICU requirement by RCS analysis. **A** Without any adjustment. **B** With adjustment of age, CCI, platelet, urea nitrogen, creatinine, and RDW

The RCS analysis was firstly performed to evaluate the association between CCI and ICU requirements. A significant nonlinear relationship (Fig. 1A) between them was observed without any adjustment (*P* = 0.017). After adjusting related variables, their nonlinear relationship was still significant (*P* = 0.008) (Fig. 1B). The results of threshold effect analysis also confirmed their nonlinear relationship (*P* = 0.003 for the LRT test). In addition, threshold effect analysis also identified a significant turning point (Table 3). Both crude and adjusted models indicated a significant association between them when CCI < turning point (all *P* < 0.001). However, their relationship was insignificant when CCI > turning point. It followed that CCI may be significantly related to the ICU admission requirement in a certain range.

**Table 3:** Threshold effect analysis between CCI and ICU requirement in patients with TAA

	Crude model		Adjusted model	
	OR (95%CI)	P	OR (95%CI)	P
Model 1				
One-line effect	0.752 (0.670, 0.844)	< 0.001	0.814 (0.700, 0.947)	0.007
Model 2				
Turning point	7		6	
< Turning point	0.504 (0.369, 0.688)	< 0.001	0.403 (0.236, 0.685)	< 0.001
> Turning point	0.960 (0.790, 1.166)	0.679	0.950 (0.790, 1.142)	0.584
LRT test		0.003		0.003

Crude: without any adjustments

Adjusted model: adjusted the age, CCI, platelet, urea nitrogen, creatinine, and RDW

Then, the independent association between CCI and ICU requirement was explored by generalized linear model (Table 4). The univariate analysis showed that all the variables were associated with the ICU requirement (all *P* < 0.05). Further, only CCI (*P* = 0.008) and creatinine (*P* = 0.022) were independently associated with the ICU requirement. Among these variables, age (*P* = 0.015) and creatinine (*P* = 0.014) showed the significant interaction effects with CCI involved in the ICU requirement.

**Table 4:** Association between CCI and ICU requirement in patients with TAA by generalized linear model

	Univariate		Multivariate	
	β (95%CI)	P	β (95%CI)	P
CCI	− 0.051 [− 0.069, − 0.033]	< 0.001	− 0.240 [− 0.417, − 0.063]	0.008
Age	− 0.005 [− 0.009, − 0.001]	0.008	− 0.009 [− 0.021, 0.002] #	0.101
Platelet	− 0.001 [− 0.001, 0.000]	0.004	− 0.001 [− 0.003, 0.002]	0.165
Urea nitrogen	− 0.007 [− 0.010, − 0.003]	< 0.001	0.002 [− 0.017, 0.021]	0.836
Creatinine	− 0.076 [− 0.123, − 0.029]	0.002	− 0.355 [− 0.660, − 0.051] #	0.022
RDW	− 0.037 [− 0.061, − 0.014]	0.002	0.003 [− 0.068, 0.073]	0.935
Marital status				
Married vs single	0.048 [− 0.072, 0.167]	0.433	− 0.009 [− 0.055, 0.036]	0.692
Divorced vs single	0.086 [− 0.097, 0.270]	0.357	0.033 [− 0.041, 0.107]	0.376
Widowed vs single	− 0.185 [− 0.360, − 0.010]	0.038	− 0.037 [− 0.111, 0.038]	0.339

^#^ means the significant interaction effects between CCI and related variables

Due to the nonlinear relationship between CCI and ICU requirement, we transformed the data of CCI as the log-CCI. After transformation, log-CCI showed a linear relationship with the ICU requirement by RCS analysis (adjusted *P* = 0.062, data not shown), which supported the logistic regression analysis. We also used logistic regression analysis to further verify the independent role of CCI in ICU requirement (Table 5). The univariate analysis showed that marital status was not associated with ICU requirement, and multivariate analysis found that only log-CCI was the independent factor of ICU requirement (*P* < 0.001). Among these variables, age (*P* = 0.023) and creatinine (*P* = 0.048) displayed the interaction effects with log-CCI.

**Table 5:** Association between log-CCI and ICU requirement in patients with TAA by logistic regression analysis

	Univariate		Multivariate	
	OR (95%CI)	P	OR (95%CI)	P
Log-CCI	0.066 [0.001,0.044]	< 0.001	0.007 [0.001, 0.108]	< 0.001
Age	0.969 [0.946,0.993]	0.010	1.018 [0.983, 1.055] #	0.317
Platelet	0.995 [0.992,0.999]	0.006	0.997 [0.993, 1.001]	0.095
Urea nitrogen	0.968 [0.951,0.986]	< 0.001	0.989 [0.958, 1.021]	0.495
Creatinine	0.696 [0.531,0.912]	0.009	1.084 [0.728, 1.614] #	0.691
RDW	0.826 [0.727,0.938]	0.003	0.980 [0.837, 1.148]	0.801
Marital status				
Married vs single	1.334 [0.656,2.711]	0.426	1.443 [0.656, 3.172]	0.362
Divorced vs single	1.760 [0.525,5.897]	0.359	2.268 [0.613, 8.391]	0.220
Widowed vs single	0.424 [0.169,1.062]	0.067	0.540 [0.178, 1.636]	0.276

^#^ means the significant interaction effects between CCI and related variables

These two models all displayed the independent role of CCI in ICU requirement, which highlighted its importance. In addition, the significant interaction effects of age and creatinine with CCI were commonly presented both in the generalized linear model and logistic regression model, which suggested the important role of age and creatinine involved in ICU requirement. These results all implied the stable association of CCI with ICU requirement even though other potential factors may affect their association.

### Clinical value of CCI in predicting ICU admission

**Fig. 2:**
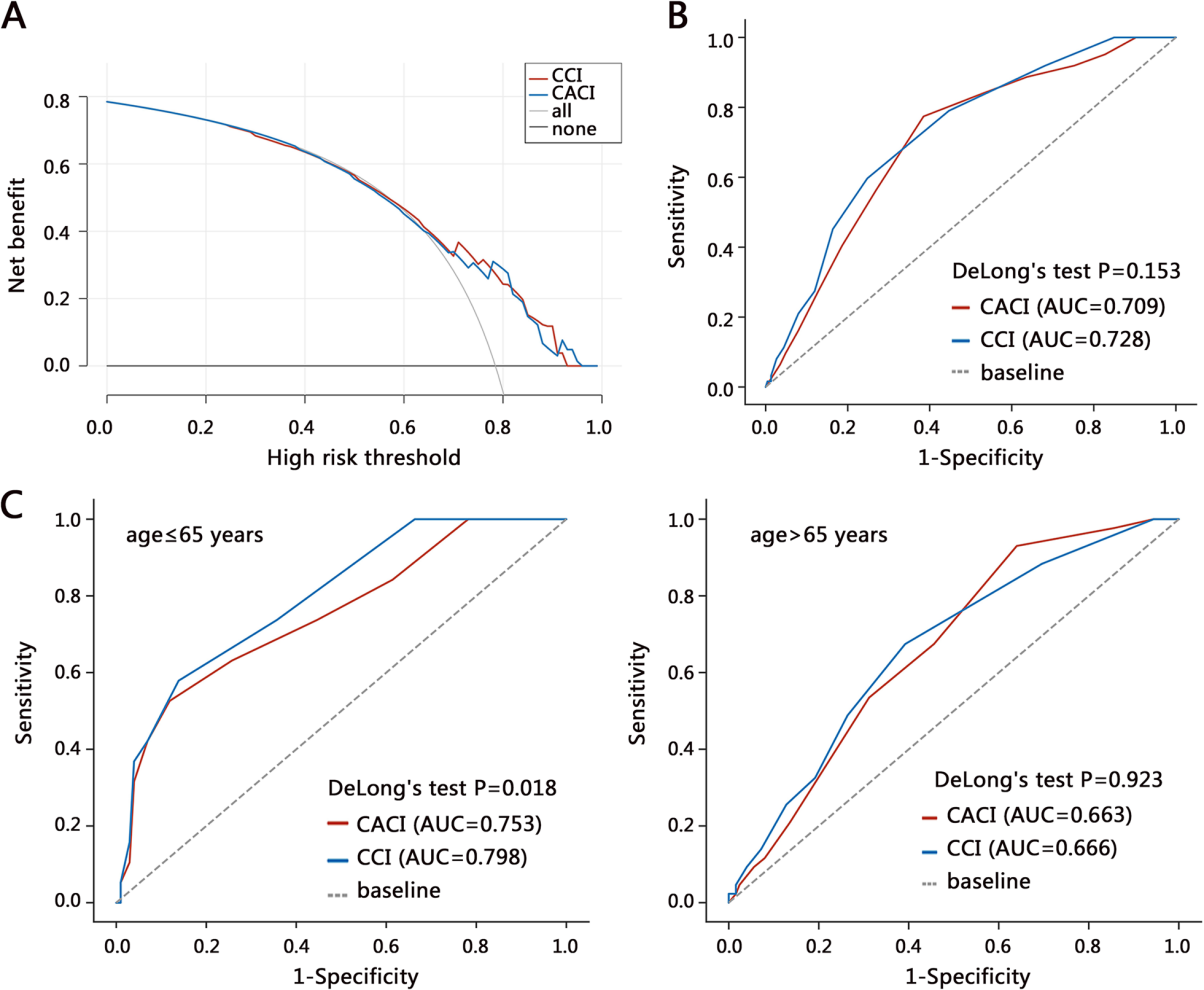
The clinical value evaluation on CCI and Charlson's age-comorbidity index (CACI) in patients with TAA. **A** The DCA and **B** ROC analyses on all patients. **C** ROC analysis on subgroups stratified by age

The above results have demonstrated the significant association between CCI and ICU admission requirement, and we further explored its potential value in clinical application. As the importance of the age-adjusted CCI (CACI) has been confirmed, therefore, we evaluated the value of CCI in predicting ICU admission compared with the CACI. CACI was calculated as the CCI after adding 1 point for each time age exceeded 40 years by 10 years (9). The DCA analysis showed that both CCI and CACI can achieve a favorable clinical net benefit for predicting the ICU requirement (Fig. 2A). ROC analysis indicated that CCI had higher AUC value than CACI (0.728 vs 0.709), though the difference was not significant (*P* = 0.153). The AUC value of CCI was also significantly higher than single age and blood variables (data not shown). In addition, a previous study has indicated that the age of 65 was related to the prediction performance of CACI in predicting ICU admission. Therefore, we also evaluated the prediction performance of CCI in subgroup patients (age ≤ 65 and age > 65) (Fig. 2C). The results showed that the predicting performance of CCI (AUC = 0.798) was significantly superior to the CACI (AUC = 0.753) in patients with age ≤ 65 (*P* = 0.018). These results highlighted that CCI may be more useful than CACI in predicting ICU admission in patients with TAA.

We further established three new models based on CCI to predict the ICU requirement. The results showed that the AUC of CCI plus age (AUC = 0.724) was similar to that of single CCI (Table 6). The AUC of two models (CCI + blood and CCI + all) was similar (AUC: 0.750 vs 0.749). Further, we used DeLong's test to compare the predicted performance of different models based on CCI. All the models were compared with the single CCI, and the *P* value of DeLong's test was all less than 0.001, suggesting the more favorable predicting performance of other models. We also performed the NRI analysis to evaluate the prediction performance of different models. The appropriate proportion of reclassification in three models was all improved (6.1%, 11.0%, and 12.6%) compared with the single CCI, indicating that the accuracy of new models was increased, and new models were superior to the single CCI for predicting the ICU requirement.

**Table 6:** The comparisons among different models for predicting the ICU requirement

		CCI	CCI + age	CCI + blood	CCI + all
ROC	Sensitivity	0.597	0.544	0.717	0.690
	Specificity	0.752	0.855	0.726	0.726
	Youden index	0.349	0.399	0.443	0.416
	AUC	0.728	0.724	0.750	0.749
	DeLong's test	/	< 0.001	< 0.001	< 0.001
NRI	Estimate		0.061	0.110	0.126
	95%CI		[− 0.01, 0.145]	[0.023, 0.205	[0.034, 0.226]
	Improvement		6.1%	11.0%	12.6%

Blood variables included RDW, platelet, urea nitrogen, and creatinine; NRI: net reclassification index

### Influence of CCI on the association between other variables and ICU requirement

Besides the importance of CCI itself in predicting ICU admission, we found that CCI may also play a vital role in the association between other variables and ICU admission. The above two regression models showed that the age, CCI, RDW, platelet, urea nitrogen, and creatinine were all related to the ICU requirement. However, most of variables showed no associations with ICU need besides the CCI in the multivariable analysis. These results may suggest the potential influence of CCI on the association between these variables and ICU requirement. In this study, we performed interaction effect analysis and mediation analysis to clarify the potential effect of CCI involved in the association between other variables and ICU requirement.

We initially performed the interaction effect analysis to explore the potential role of CCI. Based on the turning point of CCI = 6 in the adjusted model in threshold effect analysis, all the patients were divided into two subgroups (CCI < 6 and CCI ≥ 6). Then, the association between ICU requirement and these variables was explored by generalized linear model among whole populations and two subgroups (Table 7). The results showed that CCI displayed significant interaction effect on the association between ICU requirement and age, RDW, as well as platelet in all patients (all *P* < 0.001). Regarding subgroup analysis, its significant interaction effect was just observed on the association between platelet and ICU requirement in patients with CCI < 6 (*P* = 0.039). Although the interaction effect of CCI on the association between urea nitrogen/creatinine and ICU requirement was not found in whole populations (*P* = 0.384, *P* = 0.447), it was significant in CCI < 6 subgroup populations (*P* = 0.036 and *P* = 0.025). These results highlighted the important role of CCI in the association between these variables and ICU requirements, and its interaction effect may be related to the selection of a turning point. The interaction effect of CCI for these variables needs more investigations with larger samples to be verified.

**Table 7:** The interaction effect of CCI on the association between related variables and ICU requirements in patients with TAA by generalized linear model

	Subgroup	β	95%CI	P	P for interaction
Age	All	0.003	[− 0.002, 0.008]	0.199	< 0.001
	CCI < 6	0.004	[− 0.006, 0.014]	0.429	0.138
	CCI ≥ 6	0.010	[0.000, 0.019]	0.041	0.081
RDW	All	0.003	[− 0.026, 0.033]	0.816	< 0.001
	CCI < 6	0.023	[− 0.015, 0.060]	0.233	0.054
	CCI ≥ 6	− 0.012	[− 0.055, 0.031]	0.574	0.224
Platelet	All	0.001	[0.002, 2.043]	0.153	< 0.001
	CCI < 6	0.001	[− 0.001, 0.002]	0.331	0.039
	CCI ≥ 6	0.001	[− 0.002, 0.030]	0.863	0.432
Urea nitrogen	All	− 0.003	[− 0.012, 0.007]	0.592	0.384
	CCI < 6	0.008	[− 0.012, 0.028]	0.420	0.036
	CCI ≥ 6	− 0.006	[− 0.019, 0.006]	0.314	0.653
Creatinine	All	− 0.020	[− 0.172, 0.132]	0.794	0.447
	CCI < 6	0.076	[− 0.202, 0.355]	0.591	0.025
	CCI ≥ 6	− 0.141	[− 0.345, 0.062]	0.174	0.344

Further, we performed the mediation analysis to explore the mediating effect of CCI on the association between these variables and ICU requirements (Table 8). According to the total effect and indirect effect, we found that CCI showed no significant mediation effect on the association between these variables and ICU requirements after adjusting for age and four blood variables.

**Table 8:** The mediation effect of CCI on the association between related variables and ICU requirement in patients with TAA

Exposure factors	Exposure to CCI	CCI to outcome	Total effect (exposure to outcome)	Direct effect	CCI-mediated effect
Age	0.080 (< 0.001)	− 0.038 (0.001)	− 0.003 (0.114)	0.000 (0.990)	− 0.003 (< 0.001)
RDW	0.197 (< 0.001)	− 0.041 (0.002)	− 0.019 (0.130)	− 0.012 (0.361)	− 0.008 (0.032)
Platelet	0.002 (0.080)	− 0.041 (0.002)	− 0.001 (0.037)	− 0.001 (0.070)	0.000 (0.308)
Urea nitrogen	0.050 (< 0.001)	− 0.040 (0.002)	− 0.003 (0.217)	− 0.001 (0.613)	− 0.002 (0.016)
Creatinine	0.355 (0.025)	− 0.039 (0.004)	− 0.023 (0.512)	− 0.010 (0.782)	− 0.014 (0.096)

The exposure factors were age and four blood variables. The outcome was ICU requirement. The mediator was CCI. The results were presented with Coef and *P* value. All the analysis were adjusted for age and four blood variables

## Discussion

In this study, we demonstrated a significant association of CCI with ICU admission in patients with TAA who had undergone the surgery. We also confirmed the favorable clinical value and accuracy of models based on CCI for predicting ICU admission. In addition, we found that CCI exerted significant interaction effect in the association between other variables and ICU admission. Until now, there was no study reported the value of CCI involved in ICU admission prediction, and this study initially disclosed the importance of CCI in TAA.

CCI is an easy, inexpensive, and quick method of assessment. It was commonly used in different populations as a prognostic indicator in longitudinal studies to predict mortality (10, 11) and postoperative complications (12). However, little research was conducted on the prediction of ICU admission using CCI. Our study initially reported its better performance for predicting ICU admission in patients with TAA undergone the surgery. The prediction role of age-adjusted CCI in ICU admission has been revealed in other aspects. St-Louis et al. showed that the age-adjusted CCI was associated with ICU admission in the emergency general surgery population, but it was not a good predictor (13). Vahapoğlu A et al. study showed that age-adjusted CCI was effective for determining the ICU indication in the preoperative evaluation process of patients aged > 65 who had elective surgery (14). In our study, we found that CCI had a more favorable performance for predicting ICU admission than age-adjusted CCI in patients with TAA over the age of 65 who had surgery. In addition, we found that the performance of new models based on CCI was significantly increased. These results all highlighted the promising value of CCI in predicting ICU admission in TAA.

ICU admissions are usually related to serious conditions, illnesses, or complications associated with illness (15). In our study, 78.5% of patients with unruptured TAA undergone the surgery were admitted to the ICU, which may be due to the higher baseline number of patients with TAA. Hanna L et al. explored the trends in TAA hospital admissions and interventions, finding that the hospital admission incidence (mainly due to unruptured admissions) increased from 1998 (4.11/100 000) to 2020 (12.61/100 000), and operative interventions also increased mainly due to the increase in thoracic endovascular aortic repair (16). It followed that the hospital admissions of unruptured TAA and operative interventions increased objectively, which may promote ICU admission after surgery to some extent. In addition, the ICU admission was not only related to the patient’s health condition. Hicks et al. study reported that hospital-level factors including geographic location, hospital volume, teaching status, and urban/rural status, rather than patient-level factors, were the primary drivers of ICU admission after endovascular aortic aneurysm repair, and immediate ICU admission was currently overused (17). Hicks et al. study implies that it is the hospital practices that determine whether a patient needs ICU admission after surgery, whether it is warranted for the patient or not. Alshaikh et al. study found that patients admitted to the ICU not only had similar morbidity and mortality to those without admission to the ICU but also significantly correlated with increased hospital cost (18). In our study, we also found a longer length of in-hospital stay in ICU groups. The ICU is a crucial and expensive resource in the hospital and is also a special department dedicated to the most critically ill patients. Overused ICU admission and overcrowding increase morbidity and mortality (19), overworked staff, and even congestion of the patient flow as well as additional challenges in the management of ICU (20). Therefore, the requirement of ICU admission needs to be assessed reasonably and properly in preoperative.

In addition, some limitations of this study should be acknowledged. This study focused on patients with or without ICU admission. However, we cannot confirm whether these patients admitted to ICU need admission. Therefore, the false positive may be inevitable. Meanwhile, this study did not consider the ICU types, and the influence of ICU types on the prediction performance may be ignored. Though some limitations existed, this study initially reported the association between CCI and ICU admission in TAA, which provided the reference value for further investigations about the evaluation of ICU admission in patients with TAA.

## Conclusions

This study initially disclosed the significant association between CCI and ICU admission in patients with TAA undergone the surgery. The CCI is a potential predictor for evaluating the ICU admission requirement in TAA. In addition, the potential role of CCI on the association between other variables and ICU admission requirements should not be ignored.

## Data Availability

The datasets used and/or analyzed during the current study available from the corresponding author on reasonable request.

## References

[1] Lu H, Du W, Ren L, Hamblin M, Becker R, Chen Y (2021). Vascular smooth muscle cells in aortic aneurysm: from genetics to mechanisms. J Am Heart Assoc.

[2] Erbel R, Aboyans V, Boileau C, Bossone E, Di Bartolomeo R, Eggebrecht H (2015). Corrigendum to: 2014 ESC Guidelines on the diagnosis and treatment of aortic diseases. Eur Heart J.

[3] Fernando S, McIsaac D, Kubelik D, Rochwerg B, Thavorn K, Montroy K (2020). Hospital resource use and costs among abdominal aortic aneurysm repair patients admitted to the intensive care unit. J Vasc Surg.

[4] Jerath A, Laupacis A, Austin P, Wunsch H, Wijeysundera D (2018). Intensive care utilization following major noncardiac surgical procedures in Ontario, Canada: a population-based study. Intensive Care Med.

[5] Lawlor D, Lovell M, DeRose G, Forbes T, Harris K (2004). Is intensive care necessary after elective abdominal aortic aneurysm repair?. Can J Surg J.

[6] Daniels R, Nutbeam T, McNamara G, Galvin C (2011). The sepsis six and the severe sepsis resuscitation bundle: a prospective observational cohort study. Emerg Med J EMJ.

[7] Stokes P, Keheila M, Alsyouf M, Gilbert Z, Hajiha M, Amasyali A (2021). Does qSOFA score predict ICU admission and outcomes in patients with obstructed infected ureteral stones?. Can J Urol.

[8] Goulden R, Hoyle M, Monis J, Railton D, Riley V, Martin P (2018). qSOFA, SIRS and NEWS for predicting inhospital mortality and ICU admission in emergency admissions treated as sepsis. Emerg Med J EMJ.

[9] Bagni K, Chen I, Johansen A, Dehlendorff C, Jensen B, Hansen C (2020). Prognostic impact of Charlson's Age-Comorbidity Index and other risk factors in patients with pancreatic cancer. Eur J Cancer Care.

[10] Bahlis L, Diogo L, Fuchs S (2021). Charlson Comorbidity Index and other predictors of in-hospital mortality among adults with community-acquired pneumonia. Jornal brasileiro de pneumologia : publicacao oficial da Sociedade Brasileira de Pneumologia e Tisilogia.

[11] Jouffroy R, Parfait P, Gilbert B, Tourtier J, Bloch-Laine E, Ecollan P (2022). Relationship between prehospital modified Charlson Comorbidity Index and septic shock 30-day mortality. Am J Emerg Med.

[12] Hasan O, Barkat R, Rabbani A, Rabbani U, Mahmood F, Noordin S (2020). Charlson comorbidity index predicts postoperative complications in surgically treated hip fracture patients in a tertiary care hospital: Retrospective cohort of 1045 patients. Int J Surg (Lond Engl).

[13] St-Louis E, Iqbal S, Feldman L, Sudarshan M, Deckelbaum D, Razek T (2015). Using the age-adjusted Charlson comorbidity index to predict outcomes in emergency general surgery. J Trauma Acute Care Surg.

[14] Vahapoğlu A, Çavuş Z, Korkan F, Özakin O, Türkmen Ü (2023). Is a guideline required to predict the intensive care unit need of patients over 65 years of age during the pre-operative period? A comparison of the American Society of Anesthesiologists, lung ultrasound score, Charlson age-added comorbidity index, surgi. Turk J Trauma Emerg Surg TJTES..

[15] Carvalho R, Oliveira D, Pesquita C (2023). knowledge graph embeddings for ICU readmission prediction. BMC Med Inform Decis Mak.

[16] Hanna L, Sounderajah V, Abdullah A, Marshall D, Salciccioli J, Shalhoub J (2022). Trends in thoracic aortic aneurysm hospital admissions, interventions, and mortality in england between 1998 and 2020: an observational study. Eur J Vasc Endovasc Surg.

[17] Hicks C, Alshaikh H, Zarkowsky D, Bostock I, Nejim B, Malas M (2018). Intensive care unit admission after endovascular aortic aneurysm repair is primarily determined by hospital factors, adds significant cost, and is often unnecessary. J Vasc Surg.

[18] Alshaikh H, Hicks C, DiBrito S, Zarkowsky D, Siracuse J, Malas M (2019). Elective infrainguinal lower extremity bypass for claudication is associated with high postoperative intensive care utilization. J Vasc Surg.

[19] Loreto M, Lisboa T, Moreira V (2020). Early prediction of ICU readmissions using classification algorithms. Comput Biol Med.

[20] Bai J, Fügener A, Schoenfelder J, Brunner J (2018). Operations research in intensive care unit management: a literature review. Health Care Manag Sci.

